# Live imaging and quantification of circulating potentially metastatic tumor cells in early pupal stage of *Drosophila melanogaster*

**DOI:** 10.17912/micropub.biology.000588

**Published:** 2022-06-10

**Authors:** Dagem Yilma Chernet, Levi Klassen, Sonya Goertzen, Juan Nicolas Malagon

**Affiliations:** 1 Canadian Mennonite University, 500 Shaftesbury Blvd, Winnipeg, MB, Canada R3P 2N2

## Abstract

A circulating tumor cell (CTC) is a type of cell that is shed from solid tumors, swept away in the bloodstream or lymphatic system, and has the potential to cause tumorigenesis at a secondary location. Here we describe an early pupal leg system to study CTCs *in vivo* and to compare the CTCs described in this work to those previously studied *in vitro.* We quantified cellular parameters such as the number, size, and shape of CTCs, and our findings are consistent with previous *in vitro* studies. Thus, live imaging of CTCs in model organisms can complement and validate previous work in this field and can be an initial step when deciphering how *in vivo* CTCs behave in humans during metastasis.

**Figure 1. Early pupal legs as a system to studying circulating tumor cells (CTCs) in vivo.**
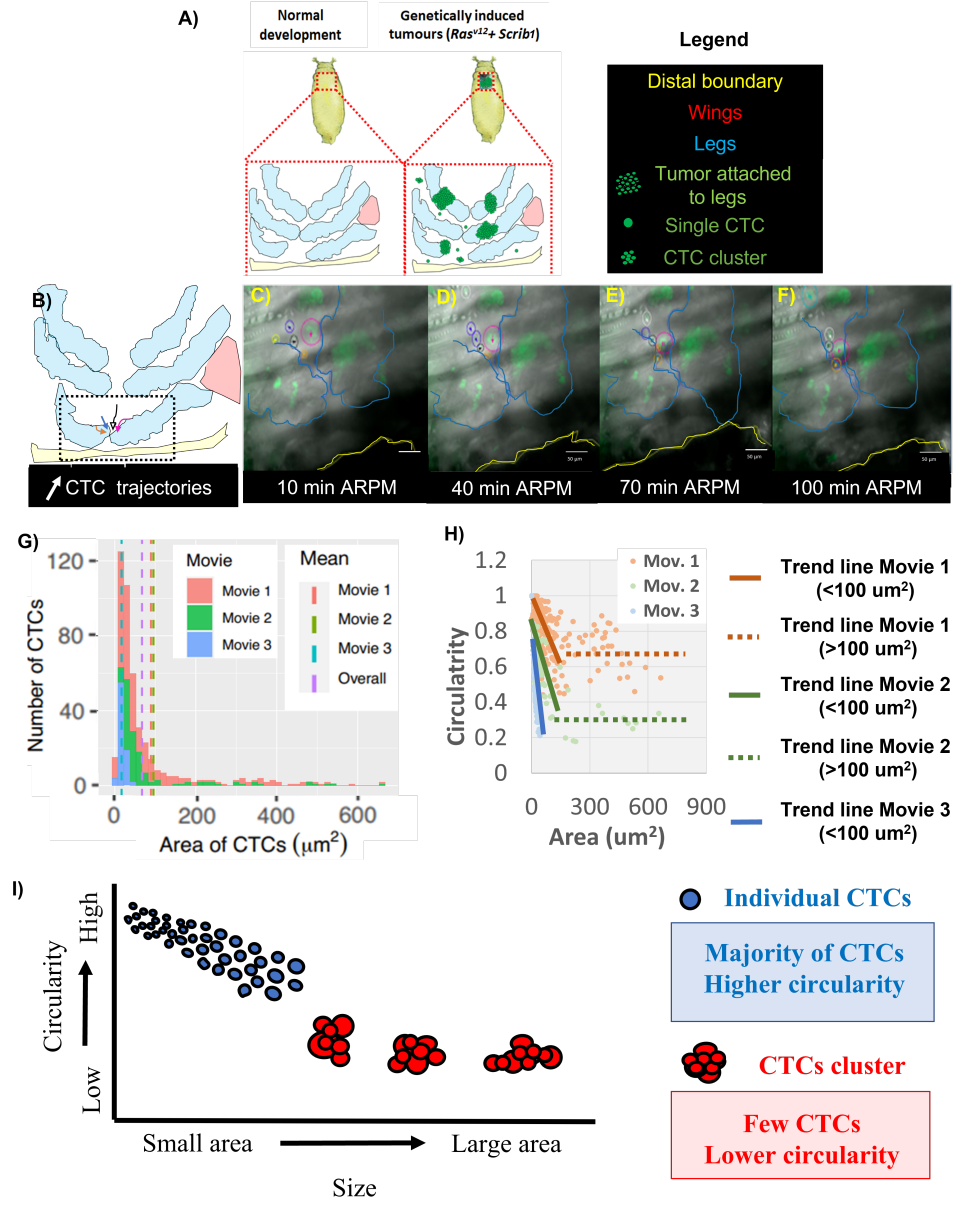
Schematic displaying developing *Drosophila melanogaster* legs of two strains: wild type vs. *Ras ^V12^ , scrib1* (genetically induced tumors). B) Schematic of developing leg with tumor and CTCs. Dotted line in B shows region imaged. C-F). Frames from Movie 1 showing CTCs movement after the rupture of peripodial membrane (ARPM) during early pupal development. In B-F, arrows indicate the direction of CTC movement. CTCs are labelled within circles of various colors. Color of legs, wings, and CTCs in the schematic A-B are matched by how they are outlined in their respective time-lapse frames. G) CTC size Histogram H) Comparison between CTC circularity and size. I) Schematic showing the relationship between CTC number, size, and circularity

## Description

Although they were discovered in the 19th century (Wang *et al.* 2017 *)* , the last 20 years have seen a renewed interest in circulating tumor cells (CTCs). CTCs originate from a primary tumor and colonize distant tissues by moving in the bloodstream (Castro-Giner and Aceto 2020). These cells may form clusters of three or more individual CTCs travelling together in the bloodstream. The relevance of CTC studies comes from the ability to detect metastasis by identifying CTC and CTC clusters in the circulatory system (Wang *et al.* 2017). Thus, understanding the biology of CTCs is important in eliminating metastatic disease by preventing their departure from tumors or destroying them while still in the bloodstream.

Despite their importance, CTCs are rare in the blood (approximately 1 CTC per 10 ^9^ blood cells) (Alix-Panabières and Pantel 2014), presenting an obstacle to those isolating and studying them (Pantel 2016). Due to this technical limitation, most work thus far has concentrated on studying CTCs *in vitro,* isolating nucleic acids from CTCs, and using antibodies to capture CTCs (Yu *et al* . 2011) *.* While these advances have been significant, the bias towards *in vitro* methods has the potential to skew analyses of CTCs towards an oversimplified understanding. The primary reasons for this are twofold. First, because the techniques used to capture CTCs rely on them showing specific cell markers, the CTCs that do not display these markers will therefore not be collected and analyzed, reducing the chances that these methods will collect a diverse set of cells (Kuo *et al.* 2019). Second, the methods which analyze CTC isolates are only able to display the properties of CTCs at a single point in time, preventing them from capturing the nuances of how CTCs can change during the metastatic process (Kuo *et al.* 2019). In addition, *in vitro* studies of CTCs can involve invasive techniques that can damage CTCs (Kuo *et al.* 2019). In contrast, *in vivo* CTC models are more likely to reproduce various aspects of the CTCs’ microenvironment including the interaction of CTCs with other cell types and tissues (Tellez-Gabriel *et al.* 2018). This technical bias raises the question of whether *in vitro* studies can adequately account for the complexity of CTCs *in vivo* , and if they do not, which exact aspects of these *in vitro* studies need to be improved.

The first step to answering this question is to develop *in vivo* systems that allow for visualizing, tracking, and reliably quantifying CTCs (Kuo *et al.* 2019). Although *Drosophila melanogaster* has an open circulatory system and cannot precisely mimic metastasis, this model organism has been used extensively to study tumor dissemination, colonization, and metastatic growth (Campbell *et al.* 2019). Here, we show that developing pupal legs in *Drosophila* are an excellent system to investigate the properties of CTCs *in vivo* . We genetically induced tumors and then performed live imaging through the pupal case, which allowed us to study CTCs without the concern of isolating, enriching, or damaging them (Kuo *et al.* 2019). In addition, although CTCs are moving along with other types of cells (e.g., immune and epithelial cells), the frequency of CTCs is much higher than in the human blood (Approximately 1 CTC per 1 other type of cell). Similar to many *in vitro* studies (Zhou *et al.* 2019), we used the image processing software *ImageJ* to quantify CTC cellular parameters and found several common patterns between our work in fruit flies and studies in human CTCs.

To find a *Drosophila* system to study CTCs, we imaged different pupal stages as well as various developing epithelia including legs, wings, mouthparts, and eyes, among others. Although we found that several epithelial systems display cell dynamics that resemble CTC formations, the presence of CTCs is infrequent and only a few CTCs can be observed. In contrast, we found that early pupal legs and wings always show the presence of CTCs at variable frequency. We imaged the following strains: normal (wt and heterozygous unflipped tissue), as well as invasive tumors ( *Ras ^V12^ ; M6 ^-/-^* ) (Dunn *et al.* 2018) and ( *Ras ^V12^ ; scrib1* ) (Wu *et al.* 2010). We observed that after the rupture of the peripodial membrane and before the head eversion, the fly strains with invasive tumor genotypes always display a presence of CTCs. In contrast, the normal strains rarely show circulating pieces of normal tissue. For this work, we are going to concentrate on *Ras ^V12^ scrib1* as this genetic combination has been studied in more detail.

Analysis of CTCs in three time-lapse movies quantified three cellular parameters: quantity of CTCs, size (measured using 2-dimensional surface area), and shape (measured using circularity). To do so, we genetically induced tumors in the larval stage and studied CTCs in the developing pupal legs. We consistently obtained GFP-labelled tumors located in the developing legs, as well as many GFP-labelled individual and groups of cells moving around them, which resemble CTCs observed in previous studies (Aceto *et al.* 2014). We found the same scenario in all pupae observed (>20 pupae), but we analyzed three movies for this study (Fig 1A-F). Then, we used the application “analyze particles” from the software *ImageJ* to compare how similar these CTCs are to those described in patients and cancer lines. In this work, we concentrated on general cellular patterns CTC, so we did not study the dynamics of each CTC over time. As a result, some of the data points belong to the same CTCs at different time points (explained in more detail in the methodology section). From these data, we observed 462 CTCs, 173 (37%) of which were CTC clusters and 289 (63%) were single CTCs. For the following measurements, individual CTCs and CTC clusters were grouped in the same category. In addition, we found high variability in the CTCs number in each movie (Fig.1G). While the average number of CTCs observed per frame in movie 1 (Mov.1) was 9.4, the other movies displayed a lower number: Mov. 2 was 4.5, and Mov. 3 was 3.6.

The size of the CTCs also varied between all three movies (Fig. 1G; Mov. 1-3). Our statistical analysis confirmed that the area datasets from each movie were significantly different from one another (Mann-Whitney U p-value is < 2.2 ^-16^ . Kruskal Wallis p-values are as follows: Mov.1 and Mov.2: 0.01846, Mov.1 and Mov.3: <2.2 ^-16^ , Mov.2 and Mov.3: <2.2 ^-16^ ). Despite these differences, 81% of the CTCs in all the movies were smaller than 100 µm ^2^ (Fig. 1G). These smaller CTCs have a far lower variation in size than the larger CTCs (<100 µm ^2^ Stdev = 21.8 = vs >100 µm ^2^ Stdev = 146.3). We also used the software *ImageJ* to investigate CTC shape. In all three of the movies, we observed a negative relationship between the area and circularity of the CTCs (Fig. 1H). Small CTCs (<100 µm ^2^ ) tend to be circular, while large CTCs (>100 µm ^2^ ) tend to have more variation in shape. However, in most the cases, CTCs display a low correlation coefficient (Mov 1 <100 µm ^2^ = 0.1749, Mov 1 >100 µm ^2^ = 0.1749, Mov 2 <100 µm ^2^ = 0.1386, Mov 2 >100 µm ^2^ = 0.0059, Mov 3 <100 µm ^2^ = 0.8774, Mov 3 >100 µm ^2^ = No CTCs higher than100 µm ^2^ were observed) (Fig. 1I), so there is only a clear linear correlation in the small CTCs in one of the movies.

Our *in vivo* findings in fruit flies show similar results to those found in human patients and cancer cell lines, and we consider that this type of work can be the first step to understanding how human CTCs behave *in vivo* . For example, as described above, we observed that small CTCs (<100 µm ^2^ ) display a lower variation in size and shape than large CTC (>100 µm ^2^ ). Similar to previous work, we observed that single CTC could be grouped in clusters, and each cluster can comprise variable numbers of single CTCs (Ligthart *et al* . 2013). As a result, the size and shape of CTC clusters can become increasingly unpredictable Fig. 1I). This phenomenon was also reported in CTC studies in breast, colorectal, and prostate cancer (Ligthart *et al* . 2013). In addition, we observed examples of CTC cluster re-organization, from a circular to a more linear configuration, resembling changes in the shape of CTCs observed when transiting through capillary-sized vessels in zebrafish (Au *et al.* 2016).

The difference in the CTC numbers and size needs to be studied in more detail. Although the videos analyzed belong to flies of a similar genetic strain and the flies were exposed to similar environmental conditions, future studies are needed to test whether this variation is a property of the system or not. Some of the variation observed among the three movies can be partially due to the difference in size and locations of tumors generated by the MARCM clones. Our current working hypothesis is that as the peripodial membrane is broken during development, the higher the number of tumors ( *Ras ^V12^* *scrib1* clones) are located in the peripodial membrane (before 6 hrs AP), the higher number of future CTCs (between 6 to 10 hrs AP).

In addition, we found a much higher percentage of CTCs cluster (37%) compared to previous work (2.6%) (Aceto *et al.* 2014). Future work needs to confirm these results using a 3-dimensional reconstruction of each CTCs because it is possible that single CTCs can appear together in our 2-dimensional projections even though they are separated. In addition, future work can also study in more detail the CTC dynamics, quantifying cell parameters such as the trajectory and velocity of CTCs.

To conclude, this work shows that using live imaging methods and multiple fluorescent markers, *Drosophila* pupal legs can be a powerful tool to study CTCs *in vivo* in a physiologically relevant system to human cancer. Furthermore, our work showed that CTCs can not only be easily tracked and quantified but also the concentration of CTCs in relationship with other cells may be much higher than those previously reported, which is one of the main problems when studying CTCs.

## Methods

Live imaging of the early pupal stages was performed between the rupture of the peripodial membrane and before head eversion. For live imaging, pupae were mounted in halocarbon oil (series 700; Halocarbon Products) on a coverslip (Sigma) and imaged with a laser 510 scanning confocal microscope (ZEISS model) at 25 degrees with a 40× objective, using LSM Browser software (ZEISS). z-stacks had a 1μm step size.

Once the time-lapses were recorded, the computer program *ImageJ* was used to analyze the videos (NIH, http://rsb.info.nih.gov/ij , released version 1.51 23 April 2018). 2D projections were generated, and every CTC or CTC cluster was manually outlined through each video. Then, the application tool “analyze particles” of *ImageJ* was used to collect information about number, area, and circularity. The dataset was analyzed using excel and R programs for graphical and statistical analysis.

To collect information about the quantity of CTCs and CTC clusters, cells were outlined in each frame of the videos. Thus, the count of CTCs obtained from this analysis does not reflect how many CTCs were present in the movies, but rather, how many were present in each individual frame of the movies. The size (area) of CTCs was measured in both individual CTCs and CTC clusters, and both types of CTCs were grouped in the same category for our analysis (Zhou *et al.* 2019). The same approach was used to analyze CTC shape (circularity).

## Reagents

Tumorigenic cells in the fruit fly pupae were generated by using tumor suppressor genes *scrib1* and oncogenes *Ras ^V12^* . Cells exhibiting *scrib1* were produced using FLP/ *FRT* -mediated clonal analysis. The cross and the progeny were kept at 18°C, and the pupae were switched to 29°C 8 hours prior to the movie and imaged at 29°C (Levayer *et al.* 2016). The tumor cells in the fruit fly legs were identified by the expression of *UAS-GFP* transgene in the *scrib1* cells. The expression of the *UAS-GFP* transgene was made possible using the MARCM system (Brumby and Richardson 2003).

**Table:** 

**Genotype**	**Source**	**Stock #**
**Wild type and unflipped clones**		
hs-flp22; ubi-Cad::GFP, UAS-mRFP; act<y+<Gal4	Levayer *et al.,* 2016	
P{ry[+t7.2]=hsFLP}22, y[1] w[*]; P{ry[+t7.2]=neoFRT}82B bon[21B]/TM3, Sb[1]	Bloomington stock center	43660
UAS-GFP.S65T; FRT82B,tub-Gal80/FRT82B	Wu *et al.,* 2010	
** Invasive clones ( *Ras ^V12^ ; scrib1) ***		
UAS-GFP.S65T/UAS-RasV12; FRT82B,tub-Gal80/FRT82B,scrib1	Wu *et al.,* 2010	
P{ry[+t7.2]=hsFLP}22, y[1] w[*]; P{ry[+t7.2]=neoFRT}82B bon[21B]/TM3, Sb[1]	Bloomington stock center	43660
** Invasive clones ( *Ras ^V12^ M6 ^-/-^ ) ***		
yw, eyFlp; Act>y+>Gal4, UAS-GFP; Tub-Gal80, FRT79E,	Dunn *et al.* , 2018	
UAS-RasV12, M6 ^-/-^ , FRT79E/TM6B		
